# Supplementary material for: Pre-Exposure Prophylaxis Interventions among Black Sexual Minority Men: A Systematic Literature Review
Source: Int J Environ Res Public Health. 2022 Feb 9;19(4):1934. doi: 10.3390/ijerph19041934 (PMC8871743; doi:10.3390/ijerph19041934)
Supplement: Supplementary file 1 [file ijerph-19-01934-s001.zip › ijerph-1555387-supplementary.pdf]

Supplement 1. Bias assessment for included studies using JBI-MASTARI instruments (n=10).

**Study Title**	**R1**	**R2**	**R3**	**R4**	**R5**	**R6**	**R7**	**R8**	**R9**	**R10**	**R11**	**R12**	**R13**
Adherence to Pre-exposure Prophylaxis in Black Men Who Have Sex with Men and Transgender Women in a Community Setting in Harlem, NY	Y	Y	Y	Y	Y	Y	Y	N	Y	Y	Y	Y	Y
A Pragmatic Randomized Controlled Trial to Increase PrEP Uptake for HIV Prevention: 55 Week Results From PrEP Chicago	Y	Y	Y	Y	Y	Y	Y	N	Y	Y	Y	Y	Y
A Randomized Controlled Pilot Study of a Culturally-Tailored Counseling Intervention to Increase Uptake of HIV Pre-exposure Prophylaxis Among Young BSMM in DC	Y	Y	N	Y	Y	Y	Y	N	Y	Y	Y	Y	Y
Embedding a Linkage to Preexposure Prophylaxis Care Intervention in Social Network Strategy and Partner Notification Services: Results from a Pilot RCT	Y	Y	Y	Y	Y	Y	Y	N	Y	Y	Y	Y	Y
Sex, PrEP, and Stigma: Experiences with HIV Pre-exposure Prophylaxis Among New York City MSM Participating in the HPTN 067/ADAPT Study	Y	Y	Y	Y	Y	Y	Y	N	Y	Y	Y	Y	Y
Small Randomized Controlled Trial of the New Passport to Wellness HIV Prevention Intervention for Black Men Who Have Sex with Men (BMSM)	Y	Y	N	Y	Y	Y	Y	N	Y	Y	Y	Y	Y
Integrated Next Step Counseling (iNSC) for Sexual Health and PrEP Use Among Young Men Who Have Sex with Men: Implementation and Observations from ATN110/113	Y	Y	Y	Y	Y	Y	Y	N	Y	Y	Y	Y	Y

**Study Title**	Q1	Q2	Q3	Q4	Q5	Q6	Q7	Q8	Q9
Social network intervention to increase pre-exposure prophylaxis (PrEP) awareness, interest, and use among African American men who have sex with men	Y	Y	Y	Y	N	Y	Y	Y	Y
Predictors of PrEP Uptake Among Patients with Equivalent Access	Y	Y	Y	N	Y	Y	Y	Y	Y
Pre-exposure prophylaxis initiation and adherence among Black men who have sex with men (MSM) in three US cities: results from the HPTN 073 study	Y	Y	Y	N	N	Y	Y	Y	Y
Integrated Next Step Counseling (iNSC) for Sexual Health and PrEP Use Among Young Men Who Have Sex with Men: Implementation and Observations from ATN110/113	Y	Y	Y	N	N	Y	Y	Y	Y

R1: Was true randomization used for assignment of participants to treatment groups?
R2: Was allocation to treatment groups concealed?
R3: Were treatment groups similar at the baseline?
R4: Were participants blind to treatment assignment?
R5: Were those delivering treatment blind to treatment assignment?
R6: Were outcomes assessors blind to treatment assignment?
R7: Were treatment groups treated identically other than the intervention of interest?
R8: Was follow up complete and if not, were differences between groups in terms of their follow up adequately described and analyzed?
R9: Were participants analyzed in the groups to which they were randomized?
Q1: Is it clear in the study what is the 'cause' and what is the 'effect' (i.e. there is no confusion about which variable comes first)?
Q2: Were the participants included in any comparisons similar?
Q3: Were the participants included in any comparisons receiving similar treatment/care, other than the exposure or intervention of interest?
Q4: Was there a control group?
Q5: Were there multiple measurements of the outcome both pre and post the intervention/exposure?
Q6: Was follow up complete and if not, were differences between groups in terms of their follow up adequately described and analyzed?
R10/Q7: Were outcomes measured in the same way between groups/timepoints?
R11/Q8: Were outcomes measured in a reliable way?
R12/Q9: Was appropriate statistical analysis used?
R13: Was the trial design appropriate, and any deviations from the standard RCT design (randomization, parallel groups) accounted for in the conduct and analysis of the trial?
